# Comprehensive Exploration of Bromophenol Derivatives: Promising Antibacterial Agents against SA and MRSA

**DOI:** 10.1021/acsomega.4c06115

**Published:** 2024-09-20

**Authors:** Ta Ngoc Ly, Le My Lan, Ming-Yu Tsai, Yun-Wen Chen, Hsin-Yi Hung

**Affiliations:** † The University of Da Nang—University of Science and Technology, Danang 550000, Vietnam; ‡ School of Pharmacy and Institute of Clinical Pharmacy and Pharmaceutical Sciences, College of Medicine, National Cheng Kung University, Tainan 701, Taiwan; § Departments of Pharmacology, College of Medicine, National Cheng Kung University, Tainan 701, Taiwan

## Abstract

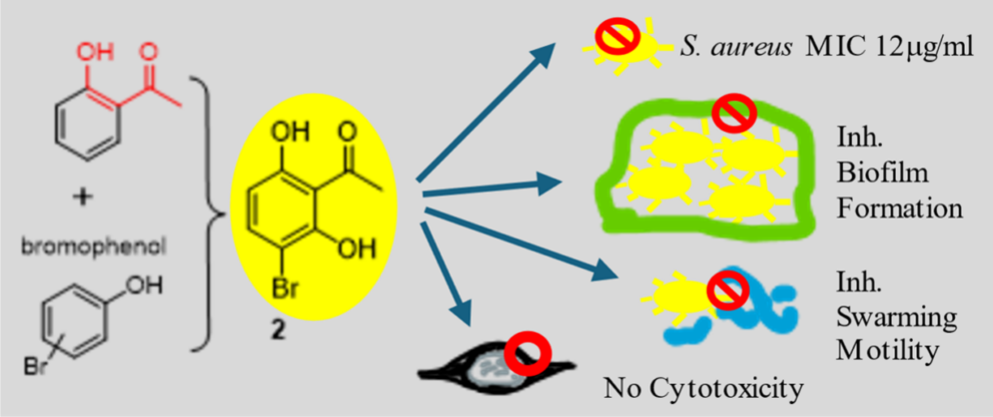

The incidence of treatment failure due to multidrug-resistant pathogens elevated over the years; the rate is much higher than new antibiotic drug discovery. Therefore, bromophenol derivatives as potential antibacterial agents on *Staphylococcus aureus* and MRSA were explored in this research via integrating chemistry, microbiology, and pharmacology to address significant knowledge gaps pertaining to the antibacterial activity of bromophenols based on their functional groups. Surprisingly, a simple molecule, 3-bromo-2,6-dihydroxyacetophenone (**2**), exhibited good anti-*S. aureus* activity and even MRSA, a drug-resistant strain. In addition, compound **2** also inhibited a common resistant pathway of pathogens, biofilm formation of *S. aureus* and MRSA. Moreover, the therapeutic index of **2** is up to 598, which can be viewed as highly selective and having low toxicity to human HEK-293 cells. Although these compounds displayed less effectiveness for the Gram-negative bacterium, *Pseudomonas aeruginosa*, they still manifested some effects on the virulence properties of *P. aeruginosa*, such as biofilm formation, pyocyanin production, and swarming motility. *In silico* analyses of the structure–activity relationship as well as ADMET properties were discussed in the end. This study shed some light on the antibacterial activities of bromophenols.

## 1 Introduction

The escalating threat of infectious diseases caused by multidrug-resistant pathogens poses a formidable challenge to global public health, with projections indicating a potential toll of over 10 million deaths annually by 2050.^1^ Amidst this background, brominated compounds have emerged as a focal point of interest within organic chemistry and pharmacology due to their diverse biological activities, particularly in the realm of antimicrobial applications.^2,3^ Bromine, positioned between chlorine and iodine in the halogen series, exhibits unique properties that confer a distinctive reactivity and potential applications to brominated compounds. In nature, marine organisms like sponges, seaweeds, and bryozoans utilize bromine chemistry to effectively control fouling.^4,5^ These organisms produce limited amounts of brominated organic compounds, which efficiently deter problematic bacteria, fungi, and algae from adhering to their surfaces, keeping them clean.^6,7^ The broader implications of brominated compounds extend to industrial applications, including their role as biocides in textiles, wooden furniture, water treatment, and other sectors.^8−10^ Of particular note are bromophenols (BPs), prevalent in marine organisms such as red algae of the *Rhodomelaceae* family, which have demonstrated significant pharmacological activities, including antimicrobial properties.^11−13^ Brominated compounds, including brominated thiophenones, furanones, and their derivatives, exhibit promising antibiofilm and antiquorum sensing activities against a range of multidrug-resistant pathogens.^14−16^ These compounds offer innovative strategies to disrupt bacterial communication and biofilm formation mechanisms, thereby attenuating virulence and enhancing susceptibility to conventional antibiotics. Continued research efforts aimed at elucidating the structure–activity relationships and in vivo efficacy of brominated compounds are warranted to harness their full therapeutic potential and address the growing challenges of antibiotic resistance in clinical settings.

This study aims to elucidate the impact of functional group modifications on the antibacterial activity of BPs, particularly focusing on their efficacy against bacterial biofilms and pyocyanin production. The unraveling of the intricate mechanisms underlying the functional group-dependent antibacterial activity of BPs provides valuable insights that could guide the development of novel antibacterial agents with enhanced efficacy and safety profiles.

## 2 Materials and Methods

### 2.1 Microbial Strains

*Staphylococcus aureus* ATCC 2793, *Pseudomonas aeruginosa* ATCC 27853, and *P. aeruginosa* PA14 were activated, cultured, and stored in a refrigerator at −25 °C in the Laboratory of Biotechnology, Faculty of Chemical, Da Nang University of Science and Technology, The University of Da Nang. The MRSA antibiotic-resistant strain was isolated, identified,^17^ and kindly provided by Dr. Ngo Thai Bich Van, Faculty of Chemical, Da Nang University of Science and Technology, The University of Da Nang.

### 2.2 Synthesis of the Tested Compounds

All chemicals were obtained from Sigma-Aldrich or Merck. The chemical reaction was monitored by thin-layer chromatography (TLC) using silica gel 60 F254-precoated glass plates with a thickness of 0.25 mm and an ultraviolet (UV) lamp to visualize the plate. Column chromatography was performed using silica gel (230–400 mesh). The NMR spectra were recorded on a Bruker AV 400 FT-NMR spectrometer.

The synthesis of compounds **1**, **2**, and **3** started from 2,6-dihydroxyacetophenone or 2,4-dihydroxyacetophenone. One equivalent of dihydroxyacetophenone was dissolved in anhydrous acetonitrile (5 mL). *N*-Bromosuccinimide, a common bromination reagent, was added to the reaction flask, and the mixture was heated at 50 °C until the starting material disappeared on the TLC plate. After removing the solvent *in vacuo*, water was added and partitioned with ethyl acetate. The ethyl acetate layer was collected and submitted to an open column packed with silica gel to obtain the desired compounds. The structures were characterized by NMR and confirmed by comparison to the literature.

3,5-Dibromo-2,6-dihydroxyacetophenone (**1**) is an amorphous yellow solid with a yield of 93%. Proton NMR signals (DMSO-*d*_6_, 400 MHz) of **1** showed δ 7.94 (s, 1H, H-4) and 2.67 (s, 3H, CH_3_).

3-Bromo-2,6-dihydroxyacetophenone (**2**) is also an amorphous yellow solid with a yield of 70%. Proton NMR signals (DMSO-*d*_6_, 400 MHz) of **2** showed δ 13.58 (br s, 1H, OH), 11.29 (br s, 1H, OH), 7.61 (d, *J* = 8.8 Hz, 1H, H-4), 6.48 (d, *J* = 8.8 Hz, 1H, H-5), and 2.71 (s, 3H, CH_3_) similar to the literature report.^18^

3,5-Dibromo-2,4-dihydroxyacetophenone (**3**) is an amorphous colorless solid with a yield of 99%. Proton NMR signals (DMSO-*d*_6_, 400 MHz) of **3** showed δ 13.39 (s, 1H, OH), 8.18 (s, 1H, H-6), and 2.66 (s, 3H, CH_3_) similar to the literature report.^19^

### 2.3 Cell Culture of HEK-293 Cells

HEK-293 cells were cultured in DMEM containing 25 mM glucose, 10% heat-inactivated fetal bovine serum (FBS), 100 U/mL penicillin, and 100 μg/mL streptomycin. Cells were incubated at 37 °C in a humidified atmosphere containing 95% air and 5% CO_2_.

### 2.4 AlamarBlue Cell Viability Assay

HEK-293 cells were seeded in 96-well plates at a density of 8000/well and incubated with the testing compounds (Table 1) at concentrations from 0.078125 to 10 μM (1:1 serially diluted). Untreated groups were set as the control (100% cell viability). On the 24th, cells were treated with the Invitrogen AlamarBlue cell viability reagent for an additional 2 h. Optical density (OD) values (at wavelengths of 570 and 600 nm) were then recorded. The percentage of cell viability was calculated according to the protocol provided by the manufacturer.

**Table 1:**
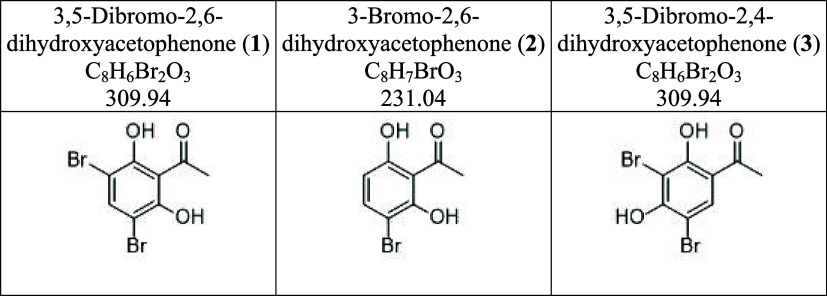
Tested Compounds

### 2.5 Calculation of Molecular Descriptors and Physicochemical Properties

The physicochemical properties essential for drug development like drug-likeness, *c* log *P*, total surface area (TSA), relative polar surface area shape index, flexibility, and complexity were calculated using DataWarrior (Version 5.2.0)

### 2.6 Scaffold Analysis

The scaffold framework was obtained by removing the terminals of all side chains attached to the ring. The analysis was performed using the Murcko scaffolds. The Murcko scaffolds were generated by eliminating the exocyclic double bonds and the α-attached atom by using DataWarrior (Version 5.2.0).

### 2.7 Similarity Analysis

The similarity between the two molecules was computed by matching flexophore descriptors derived from the molecular structure. This involved creating a representative range of conformers.

### 2.8 Activity Cliff Analysis

Activity cliffs are pairs of compounds with high structure similarity but significantly different biological activity. The structure–activity landscape index (SALI) is widely used in chemoinformatics. SALI maps are one of the approaches to studying activity landscapes and rapidly identifying activity cliffs. The identification of activity cliffs assumes significance, as it elucidates specific and minor modifications within molecular structures that exert a profound influence on biological activity. In this investigation, the detection of potential activity cliffs is facilitated through the computation of structural similarities and disparities. The analysis employs DataWarrior to discern and assess the presence of activity cliffs, with due consideration to the possibility of measurement inaccuracies influencing the data set. The map displayed structure similarity on the *X*-axis. The *Y*-axis showed the activity difference using biological activity values of tested compounds. The data points in the maps were further colored by their SALI value. The SALI values were calculated based on activity cliff analysis correlating the biological properties with the chemical properties of tested compounds.

### 2.9 *In Vitro* Antibacterial Screening

The susceptibility test was measured *in vitro* by employing the agar well diffusion method.^20^*S. aureus* ATCC 2793 and *P. aeruginosa* ATCC 27853 strains were used in this experiment. A suspension of the test organism was prepared in nutrient broth by overnight culture for 24 h at 37 °C and 7.0 pH. The agar plate surface was inoculated by spreading a volume of the microbial inoculum over the entire agar surface. Then, a hole with a diameter of 6 to 8 mm was punched aseptically with a sterile tip, and a volume (20 μL) of the tested solution at the desired concentration was introduced into the well. Then, agar plates were incubated under suitable conditions depending upon the test microorganism. After incubation, the plates were examined and the diameters of the zone of complete inhibition were observed.

The antibacterial efficacy of the chosen compounds was assessed through the microbroth dilution assay conducted in 96-well culture plates.^21^ A stock solution with a concentration of 20 mg/mL was prepared in dimethyl sulfoxide (DMSO). Subsequently, autoclaved nutrient broth (100 μL) was introduced into the culture plate wells, with the initial row of the microtiter plate receiving 100 μL of the test material. 2-fold serial dilutions of the test compounds were then meticulously executed. To serve as an indicator, 20 μL of 2× resazurin solution was added to each well. Finally, 10 μL of the bacterial suspension was incorporated into each well, yielding a final concentration of 5 × 10^6^ CFU/mL. The experiment was conducted in duplicate, and the plates were incubated at 37 °C. After 18 h, the plates were scrutinized for any change in color indicative of bacterial growth. The compound was deemed active if the wells exhibited clarity without discernible bacterial growth, and the outcome was quantified as the minimum inhibitory concentration (MIC).

### 2.10 Biofilm Formation Assessment

Biofilm formation was evaluated utilizing the crystal violet assay.^22^ The *P. aeruginosa* ATCC 27853 strain was used in this experiment. The bacterial attachment was initiated by inoculating the culture medium with approximately 10^7^ CFU/mL in polystyrene 96-well plates, followed by incubation at 37 °C for 2 h in aerobic conditions. Subsequent to the attachment phase, the medium was carefully aspirated, and the 96-well plates were rinsed with phosphate-buffered saline (PBS) (pH 7.0) to eliminate unattached cells. The fresh medium, along with tested compounds, was then introduced to assess the inhibitory impact on biofilm formation. Following an 18 h incubation at 37 °C, a crystal violet (CV) assay was conducted. The well plate was washed with PBS to eliminate unattached cells, and a 1% CV solution was added, allowing for a 30 min incubation at room temperature. After this period, the well plate underwent three PBS washes, and absolute ethanol was added, incubating for 15 min. Following the transfer of the stained solution to a new well plate, the absorbance was measured at 595 nm.

### 2.11 Pyocyanin Inhibition Assay

Pyocyanin quantification was conducted utilizing the chloroform extraction method.^23^*PA14* was subjected to incubation with the tested compounds at concentrations corresponding to half of the minimum inhibitory concentration (MIC) for a duration of 12 h. Following the specified incubation period, the cultures underwent centrifugation at 10,000 rpm for 10 min, leading to the collection of the supernatant. Subsequently, 3 mL of chloroform was added to 5 mL of the supernatant, and the mixture was vortexed for 20 s. After centrifugation at 5000 rpm for 10 min, the resulting blue layer at the bottom was transferred to a new tube. To each tube, 2 mL of 0.2 M HCl was added and vortexed for 20 s. The sample underwent subsequent centrifugation for 2 min at 5000 rpm, and 1 mL of the resulting pink layer was transferred to cuvettes. A blank was established using 0.2 M HCl. Spectrophotometric measurements were carried out at 520 nm.

### 2.12 Swarming Motility

The analysis was performed in Petri dishes with the LB minimal medium supplemented with 0.2% glucose and 0.5% agar, as described by Kearns.^24^ Tested compounds were added to the motility agar at the desired concentration. Aliquots (1.0 μL) were taken from *P. aeruginosa* ATCC 27853 overnight cultures and spotted in the center of each well, and the migration zones were measured after 24 h of incubation at 37 °C. The inhibition rate was calculated as follows:
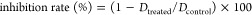
where *D*_treated_ and *D*_control_ are the migration distances in the treated and control samples, respectively.

### 2.13 Statistical Analysis

All experiments for antibacterial activities were carried out in triplicate, and a one-way ANOVA was performed to examine the effect of compounds on bacteria using Microsoft Excel. For the analysis of biofilm formation inhibition and Pyocyanin production inhibition results, treated groups were compared with the control using a one-way analysis of variance and a post-hoc Tukey’s test. The p-value of 0.05 was considered statistically significant.

## 3 Results and Discussion

### 3.1 Chemistry

**Figure 1:**
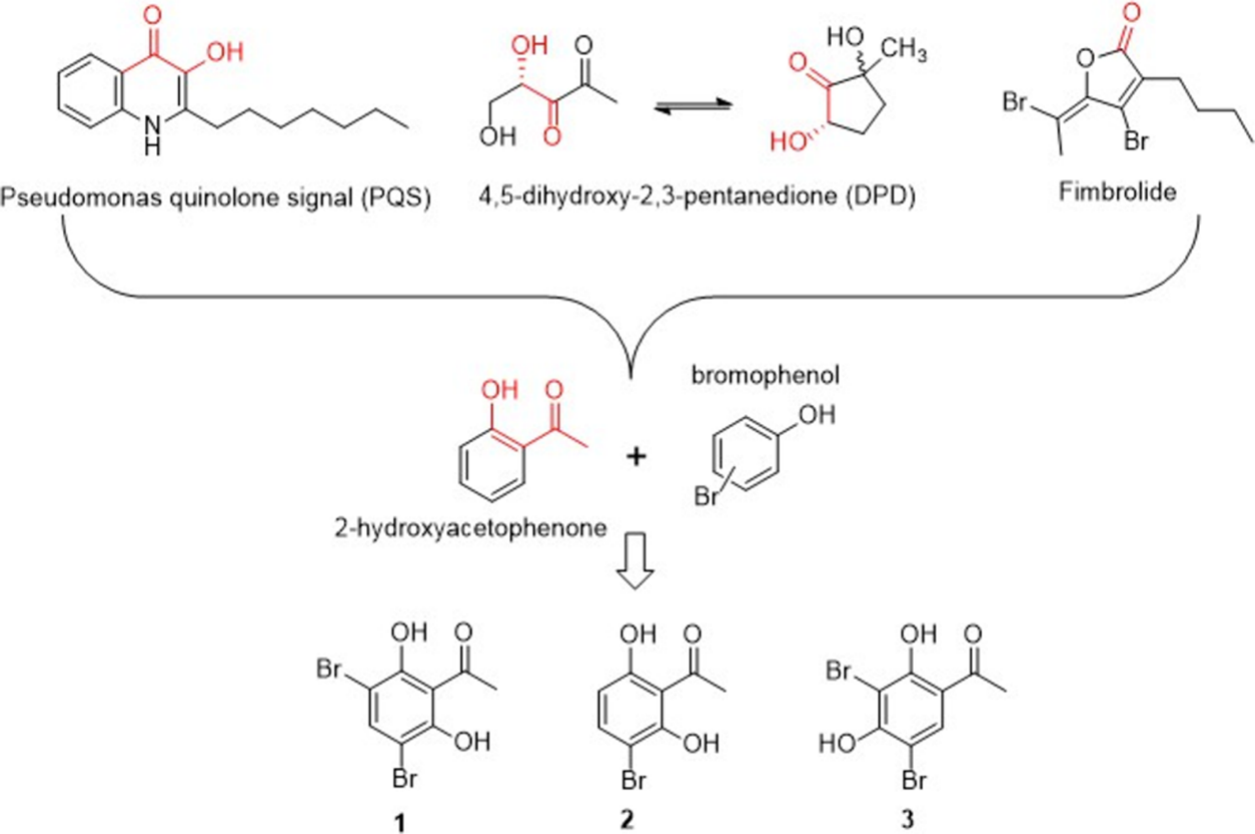
Rationale design of BPs.

Since bromophenols exhibit good antibacterial and antibiofilm activity, the basic skeleton was set to include bromo and phenol groups. In addition to the bromophenol skeleton, a series number of literature regarding antibiofilm formation was reviewed and an interesting finding emerged (Figure 1).^25,26^ When the structures of the important signal molecules or known antibiofilm formation inhibitors, such as the pseudomonas quinolone signal (PQS),^26^ 4,5-dihydroxy-2,3-pentanedione,^27^ and fimbrolide,^28^ were examined, a ketone group accompanying a hydroxy group frequently showed up and it seems to be crucial to the activity. A bold hypothesis came to our mind to incorporate 2-hydroxyacetophenone with bromophenol to see whether this simple molecule is active against bacteria or not. Moreover, based on our previous study (unpublished data) and the literature,^26,27,29^ dihydroxy substitution exhibited better activity than monosubstitution. Thus, with 2-hydroxyacetophenone as the basic skeleton, different substitutions of bromo and hydroxy groups were designed and synthesized.

The synthesis of compounds **1**, **2**, and **3** was performed using a common brominated reagent, *N*-bromosuccinimide in acetonitrile with the corresponding starting materials, heated to 50 °C until the starting material disappeared. Mono- or dibromo products could be obtained but were separable on silica gel columns. Characterization of the compounds was performed using NMR spectroscopy and confirmed by comparison to the literature. Other substitution patterns, such as bromo-2,5-dihydroxyacetophenone, cannot be obtained due to the instability of the product. Finally, only compounds **1, 2**, and **3** were sent for bioactivity evaluations.

### 3.2 Antibacterial Activity on *S.aureus* and *P.aeruginosa*

First, the antibacterial capabilities of bromophenol derivatives were tested using the agar diffusion method. The results (Table 2) demonstrate that our compounds exhibit significant antibacterial activity against the tested bacterial strains. The antibacterial activity of compounds **1**–**3** has a pronounced effect on *S. aureus* and MRSA, producing the largest antibacterial zone diameter with compound **2**. However, these compounds exhibited fewer effects on *P. aeruginosa*. Ampicillin exhibited a significant zone diameter of 20 mm against SA, indicating considerable antibacterial activity. In contrast, there was no observable zone diameter when ampicillin was tested against PA. The observed zone diameters for tobramycin against *S. aureus* and MRSA were 15 and 12 mm, respectively. In the assessment of the antibacterial effectiveness of tobramycin and tetracycline against *P. aeruginosa*, the observed zone diameters were 15 and 10 mm. These findings deviate slightly from the standardized data provided by the Clinical Laboratory Standards Institute, wherein the expected zone diameters for tobramycin and tetracycline against SA are reported to be within the range of 12–14 mm,^30^ respectively. The plausible reason is that for *S. aureus* and MRSA, Gram-positive bacteria with only a peptidoglycan layer, the compounds can easily diffuse into the cells and destroy the bacteria. On the other hand, for *P. aeruginosa*, a Gram-negative bacterium with a thicker cell wall and an additional layer of lipopolysaccharide, the ability to diffuse into the bacterial cell is significantly reduced. Interestingly, the antibacterial activity results for antibiotic-resistant MRSA show higher activity compared to regular *S. aureus*.

**Table 2:** Diameter of Zone Inhibition of Tested Compounds and Antibiotic Standards

		inhibition zone diameter (mm)		
compounds/antibiotics	concentration (μg)	*S. aureus*	MRSA	*P. aeruginosa*
**1**	20	26	28	2
**2**	20	29	30	1
**3**	20	12	20	1
ampicillin	10	16	18	
tobramycin	10	15	12	15
tetracycline	30	14	12	10

To gain further insights into the inhibitory potential of the compounds, a survey of the MIC for these three compounds was conducted (Table 3) along with the comparison to other BPs (compounds **4**–**8**) published by Ming Liu and colleagues.^7^ One of the five BPs isolated from the seaweed *Rhodomela confervoides* (bis(2,3-dibromo-4,5-dihydroxybenzyl)ether) had the strongest activity with the MIC of 35 μg/mL on *Staphylcoccus epidermidis*, while the other BPs showed moderate activity with the MIC in the range of 70–140 μg/mL on *S. aureus*. Ampicillin showed the MIC of 10 μg/mL against *S. aureus* and was inactive against *P. aeruginosa*, while tetracycline displayed the MIC of 30 μg/mL against SA and 70 μg/mL against *P. aeruginosa*. Tobramycin exhibited the MIC of 25 μg/mL against *S. aureus* and 15 μg/mL against *P. aeruginosa*. Although compounds **1** and **2** manifested MIC values lower than two antibiotics tetracycline and tobramycin, they were higher than the MICs of ampicillin, showing good antibacterial ability, proving that they have the potential to develop into antibiotics.

**Table 3:** Antibacterial Activity of Compounds **1**–**3**, Bromo Benzenediol Derivatives, and Antibiotics

	MIC (μg/mL)	
tested compounds	*S. aureus*	*P. aeruginosa*
**1**	24	780
**2**	12	780
**3**	390	780
3-bromo-4-(2,3-dibromo-4,5-dihydroxyphenyl) methyl-5-(hydroxymethyl)-1,2-benzenediol (**4**)^a^	140	>140
3-bromo-4-(2,3-dibromo-4,5-dihydroxyphenyl) methyl-5-(ethoxymethyl)-1,2-benzenediol (**5**)^a^	70	>140
3-bromo-4-(2,3-dibromo-4,5-dihydroxyphenyl) methyl-5-(methoxymethyl)-1,2-benzenediol (**6**)^a^	70	>140
4,4′-methylenebis(5,6-dibromo-1,2-benzenediol) (**7**)^a^	140	140
bis(2,3-dibromo-4,5-dihydroxybenzyl)ether (**8**)^a^	70	70
ampicillin	10	
tetracycline	30	70
tobramycin	25	15

a Bromo benzenediol derivatives from the study of Liu et al.^7^

### 3.3 Biofilm Formation Inhibition of BP Derivatives on *S. aureus* and MRSA

Biofilm formation is an important tool for bacteria to overcome antibiotics. Therefore, three BP derivatives were tested on the inhibition of *S. aureus* and MRSA biofilm formation in the MIC and 1/2 MIC concentrations (Table 4). All three compounds can inhibit biofilm formation. Among them, compound **2** showed the highest inhibition rate on *S. aureus*, which is completely compatible with the results of the antibacterial activity test mentioned above. In addition, it can be proven that DMSO can inhibit biofilm formation on microorganisms, which may disturb the results and has been proven by Summer and colleagues^31^ about the impact of DMSO at concentrations of 0.03–25% on inhibiting membrane formation on *S. aureus*. Thus, the tested compounds’ groups exhibited significant biofilm formation inhibition, much more than the vehicle group containing DMSO, showing that our compounds indeed displayed an ability to inhibit biofilm formation.

**Table 4:** Biofilm Formation Inhibition of Compouds **1**–**3** on *S. aureus*^a^^b^

	biofilm formation inhibition (%)			
	**1**	**2**	**3**	vehicle^b^
MIC	81.55* ± 0.79	84.79** ± 0.38	75.67* ± 2.91	42.90 ± 1.73
1/2 MIC	79.10* ± 1.12	79.41* ± 0.39	67.61^ns^ ± 2.54	36.17 ± 1.83

a Values are presented as mean ± SD with *n* = 3. Degrees of significance determined using an ANOVA with a post-hoc Tukey’s test at a 5% significance level to compare the control with the vehicle are * = highly significant and ns = no significant difference.

b Vehicle contains 10% DMSO.

### 3.4 BP Derivatives Exhibit Antivirulence Properties in *P. aeruginosa*

**Figure 2:**
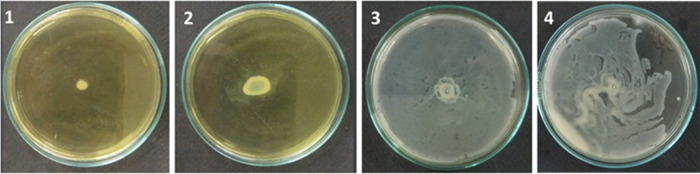
Effects of compounds (**1**–**3**) and the control (**4**) on the swarming motility of *P. aeruginosa*.

*P. aeruginosa* produces several virulence factors, such as pyocyanin, positively regulated by quorum sensing. Here, the BPs’ effects on pyocyanin production in the PA14 strain and the swarming motility in the PA27853 strain were analyzed (Tables 5 and 6 and Figure 2).

**Table 5:** Antivirulence Properties of Compounds **1**–**3** on *P. aeruginosa*^a^^b^

	**1**	**2**	**3**	vehicle^b^
biofilm formation inhibition (%)	22.26** ± 7.90	14.27* ± 0.41	14.55 ^ns^ ± 1.06	4.03 ± 0.27
pyocyanin production inhibition (%)	68.82* ± 13.35	52.51* ± 3.82	38.92^ns^ ± 9.77	9.36 ± 1.40

a Values are presented as mean ± SD with *n* = 3. Degrees of significance determined using an ANOVA with a post-hoc Tukey’s test at a 5% significance level to compare the control with the vehicle are * = highly significant and ns = no significant difference.

b Vehicle contains 10% DMSO.

**Table 6:** Swarming Motility Inhibition Activity in *P. aeruginosa* of Compounds **1**–**3**

	**1**	**2**	**3**	control
migration distance (mm)	6.5	13.5	13.5	100
inhibition rate (%)	93.5	86.5	86.5	0

Compound **1** demonstrated the highest biofilm inhibition (22%) and significant pyocyanin production inhibition (68%), suggesting strong antibiofilm and antiquorum sensing activities on *P. aeruginosa*. Compound **2** exhibited moderate biofilm inhibition (14%) and pyocyanin production inhibition (52%), while compound **3** showed similar biofilm inhibition (14%) but lower pyocyanin production inhibition (38%). In the swarming motility test, we observed significant differences in the migration distances of *P. aeruginosa* under different treatment conditions. The migration distance was 6.5 mm in the medium containing compound **1** and 13.5 mm in the presence of compound **2** or compound **3**. The inhibition rates for compounds **1**, **2**, and **3** were 93.5, 86.5, and 86.5%, respectively, indicating strong inhibitory effects on the swarming motility of *P. aeruginosa*. These results suggest that compound **1** exhibits the most potent inhibition of *P. aeruginosa* swarming, while compounds **2** and **3** also significantly hinder bacterial mobility. Compound **1** may interfere with the normal functioning of the swarming motility mechanism, preventing the bacteria from spreading rapidly across surfaces. The results highlight compound **1** as a promising candidate for further development due to its potent antibiofilm and antiquorum sensing properties against *P. aeruginosa*, with compounds **2** and **3** showing the potential for further chemical optimization.

### 3.5 Toxicity of Tested Compounds

**Figure 3:**
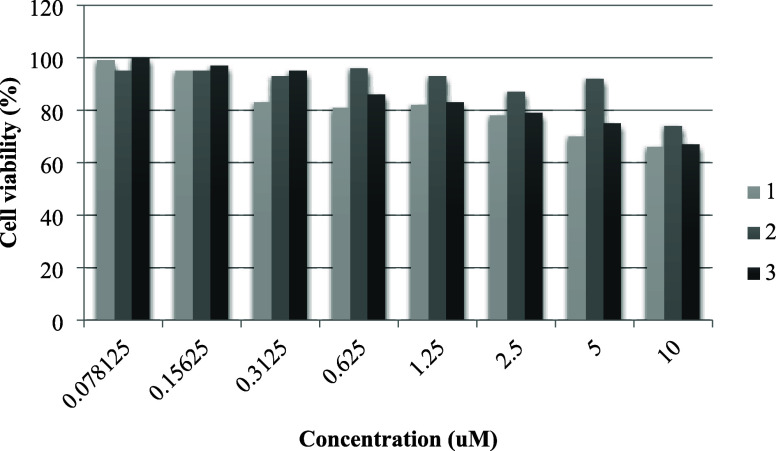
Toxicity test on HEK-293.

The assessment of the BPs’ toxicity was conducted through the evaluation of cell viability in HEK-293 cells exposed to varying concentrations. Initial exposure involved doses ranging from 0.07 to 10 μM to establish the IC_50_ value for HEK-293 cells (Figure 3).

Post-treatment revealed a dose-dependent decline in cell viability, with IC_50_ values for compounds **1**, **2**, and **3** measured at 14.8, 31.1, and 32.0 μM, respectively. Notably, compound **1** exhibited a more pronounced inhibitory effect on cell growth at lower concentrations compared to compounds **2** and **3**. The calculation of the therapeutic index (Table 7), represented by the cytotoxicity IC_50_ (μM)/MIC (μM), yielded values of 191 for compound **1**, 598 for compound **2**, and 25 for compound **3**. Toxicity evaluation on HEK-293 cells indicates that compound **2** exhibits excellent selectivity in its cytotoxicity to *S. aureus* strains, laying the groundwork for further safety assessments and potential therapeutic development.

**Table 7:** IC_50_ and TI

		therapeutic index (IC_50_/MIC)^a^	
tested compounds	HEK-293 IC_50_ (μM)	*S. aureus*	*P. aeruginosa*
**1**	14.8	191	4
**2**	31.1	598	3
**3**	>32.0	25	2

a Therapeutic index is calculated by cytotoxicity IC_50_(μM)/MIC(μM).

### 3.6 Physicochemical Property Analysis

Physicochemical (PC) properties play a crucial role in influencing drug efficacy, safety, and metabolism. Small-molecule drug candidates need to be both soluble and permeable for experimental assays and effective targeting. Understanding PC parameters can aid in designing compounds with multiple biological targets and polypharmacology profiles, beneficial for treating diseases with complex origins. To evaluate the quality of research compounds, linking potency, and lipophilicity in an attempt to estimate drug-likeness, the chemical properties such as polar surface area, ligand efficiency, and shape index were calculated and are shown in Table 8.

**Table 8:** Physicochemical Property *In Silico* Analysis

tested compounds	*c* log *P*	total surface area	relative PSA	ligand efficiency	lipophilic ligand efficiency	shape index	molecular flexibility	molecular complexity
**1**	2.28	153.23	0.25	0.82	5.5	0.46	0.36	0.79
**2**	1.56	134.6	0.29	0.85	5.9	0.50	0.38	0.74
**3**	2.28	153.23	0.25	0.79	5.2	0.53	0.31	0.76

Lipophilicity, characterized here by computed *c* log *P* values, plays a crucial role in determining several ADMET parameters as well as potency. The surface area and the related topological surface area (TPSA) are other commonly investigated descriptors related to hydrogen bonding that is important for permeability estimation and oral bioavailability. Specifically, the *c* log *P* and the surface area of compound **2** were notably less than those of compounds **1** and **3**. Notably, a molecule’s TPSA of compound **2** was notably higher than those of compounds **1** and **3**, indicating the difference in hydrogen bonding in compound **2**.

The ligand efficiency gauges the impact of a compound’s heavy atoms or its molecular weight on its potency or binding affinity. Essentially, it measures potency or binding affinity per heavy atom or molecular weight. Compound **2** demonstrated a ligand efficiency (LE) of 0.85, outpacing the values of **1** and **3**, which stood at 0.82 and 0.79. Meanwhile, the ligand lipophilicity efficiency (LLE) links potency or binding affinity with lipophilicity. It evaluates how adeptly a compound leverages its lipophilicity for binding or potency without excessive lipophilicity. Compound **2** showcased a notably superior LLE value (5.9) compared to **1** and **3** (5.5, 5.2). Given that an LLE value above five is considered optimal, compound **2**’s result suggests that its hydrophobic region optimally interacts with biological targets, influencing its observed bioactivity. A higher LLE indicates how well a compound leverages its lipophilicity for binding or potency without excessive lipophilicity. Compound **2**’s higher LLE suggests an optimal interaction with biological targets, contributing to its observed bioactivity against SA.

The shape index, as computed using Datawarrior, evaluates the three-dimensional (3D) configurations of compounds. A shape index below 0.5 indicates 3D or spherical structures, while a value above 0.5 suggests flat structures. Compound **3** displayed a shape index of 0.53, exceeding those of compound **2** (0.50) and compound **1** (0.46). This implies that compound **3** comprises flat structures, whereas compounds **1** and **2** possess more rounded or 3D structures. The 3D configurations of compounds play a role in their bioactivity. The flatter structure of compound **3** might influence its interaction with biological targets, impacting bioactivity differently compared to more 3D structures of **1** and **2**.

The flexibility plays a crucial role in oral bioavailability, and in the early stages of drug discovery, it is frequently quantified by the number of rotatable bonds. The unhindered rotation of atoms around these single bonds allows the molecule to adopt various conformations, and an increase in the number of single bonds corresponds to heightened flexibility. Molecular complexity is another property known to influence events such as solubility, oral bioavailability, permeability, promiscuity, and clinical progression. This measure accounts for the number of rings and aromatic rings, the fraction of carbons that are sp^3^-hybridized, or the number of stereocenters. The complexity of a molecule significantly influences its affinity for and specificity against targets. In our analysis, compound **2** exhibits a higher flexibility score and a lower complexity score compared to compounds **1** and **3**.

Molecular flexibility, influenced by the number of rotatable bonds, can affect oral bioavailability. Higher complexity may influence specificity against targets.

A distinct connection exists between attributes such as the LE, LLE, shape index, molecular flexibility, and bioactivity of tested compounds. Specifically, compound **2** showcased notable anti-SA activity and effectively inhibited biofilm formation. Given these insights, considering logP and binding efficiency metrics like the LE and LLE is crucial not just for initial selection but also for subsequent lead optimization and generation.

### 3.7 Activity Cliff and Structure–Activity Relationship (SAR) *In Silico* Analysis

An “activity cliff” refers to pairs of compounds that share structural similarities but show significant disparities in bioactivity or potency. This phenomenon has captivated both medicinal and computational chemists over the years. Utilizing DataWarrior, a structure–activity similarity analysis was performed.

**Figure 4:**
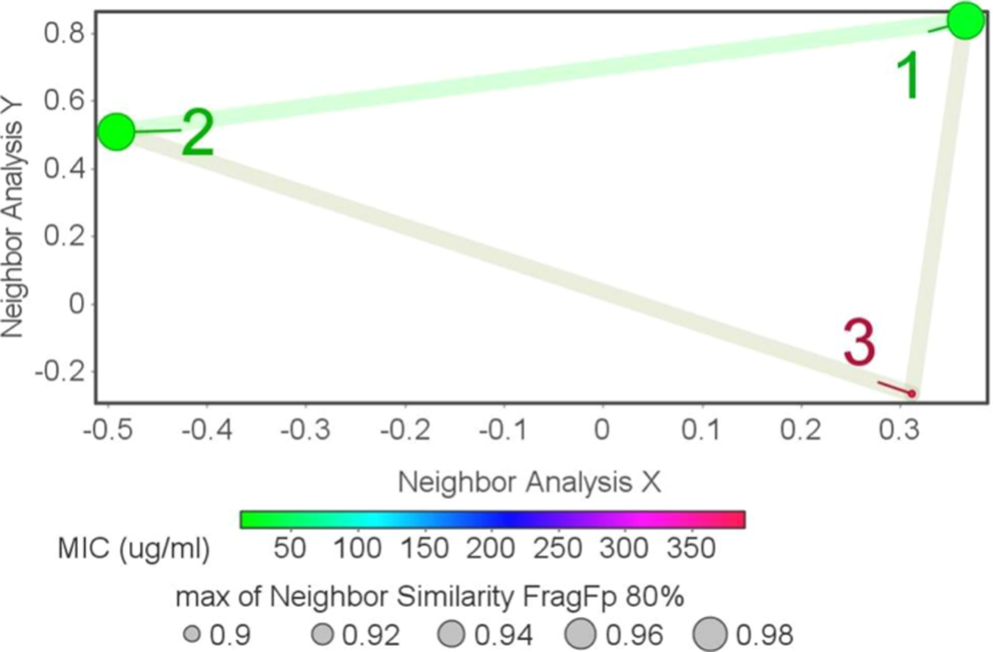
Structure–activity landscape index (SALI) plot.

Similarity analysis reveals a high level of similarity among the three compounds, with compounds **1** and **2** being the most similar, as indicated by the pink line connecting them. These color-coded markers signify comparable activity levels. Within these clusters of tested compounds, structural resemblances juxtaposed with varied bioactivities were observed, exemplifying classic activity cliffs (Figure 4).

The presence of activity cliffs underscores the importance of subtle structural changes. Functional groups such as bromo, ketone, and hydroxyl play a role in influencing bioactivity, and the identification of activity cliffs provides insights for further structure–activity relationship (SAR) investigations.

### 3.8 SAR Analysis

**Figure 5:**
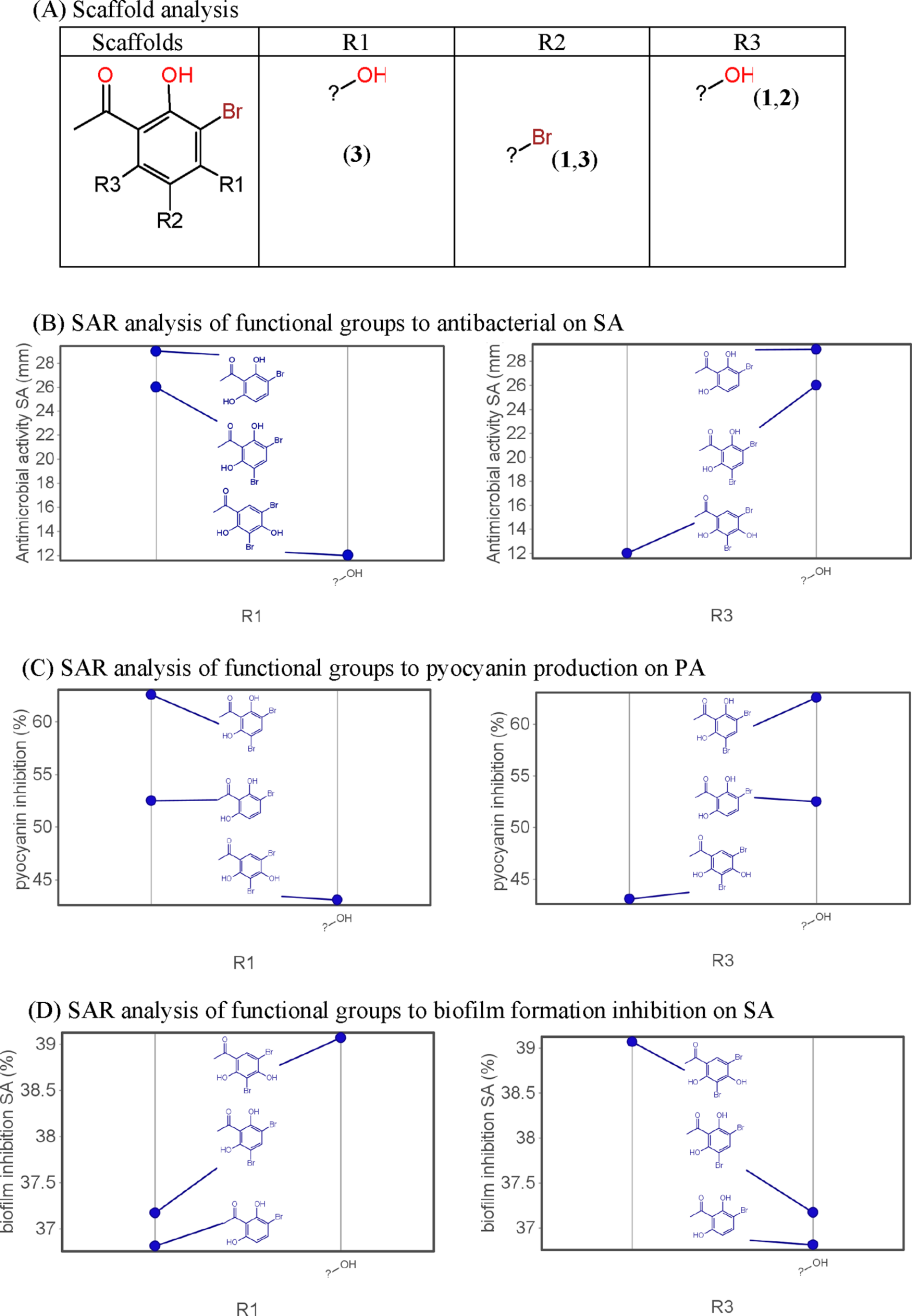
Scaffold and structure–activity relationship (SAR) of BPs’ derivatives.

SAR analysis emphasizes the influence of functional group attachments on bioactivity, as shown in Figure 5. Bromo, ketone, and hydroxyl groups significantly impact antibacterial and quorum sensing (QS) inhibitory activities. The position and adjacency of hydroxyl groups, as well as the presence of bromo groups, are crucial for modulating bioactivity.

Antibacterial activity illustrates that the bromo group significantly affects the antibacterial ability of the compound. When comparing the activities of **1** and **2**, adding a bromo group to the para position shows a significant difference in activity. Compound **1** with two bromo groups yields a higher activity compared to attaching one bromine group to the ring. However, this result contradicts the anti-*S. aureus* activity where one bromo group yields a higher activity than two bromo groups. Specifically, bromo-containing compounds exhibit a substantial increase in their ability to inhibit biofilm formation by up to 22% and demonstrate a remarkable 66% inhibition in pyocyanin. Bromo substitution significantly increases the ability to inhibit QS through the inhibition of biofilm and pyocyanin production.

SAR analysis revealed distinct patterns regarding the impact of hydroxyl groups at different positions on the bioactivity of the compounds. In particular, the presence of a hydroxyl group in R1 was found to reduce the resistance to SA (Figure 5B). Conversely, the hydroxyl group in R3 was associated with an increase in SA resistance, suggesting a contradicting effect (Figure 5B). In the context of pyocyanin inhibition in *P. aeruginosa*, the hydroxyl group in R3 was found to enhance inhibition, whereas its presence in R1 decreased pyocyanin inhibition in PA (Figure 5C). In *S. aureus*, the hydroxyl group in R3 was associated with a reduction in biofilm formation, while the opposite effect was observed with the hydroxyl group in R1 (Figure 5D). The position of the hydroxyl group on the benzene ring is a decisive factor in the activity expression. When the other hydroxyl group is adjacent to the ketone group, it exhibits a stronger activity than when it is located elsewhere. This finding aligns with previous research by Ming and colleagues.^7^ Summing up the *in silico* analysis, compound **2** exhibited favorable properties, including a good oral absorption potential, ligand efficiency, and optimal interaction with biological targets.

### 3.9 Absorption, Distribution, Metabolism, Excretion, and Toxicity *In Silico* Analysis

To evaluate the absorption properties of the tested compounds, human intestinal absorption (HIA), Caco-2-Permeability, and *P*-glycoprotein substrates or inhibitors were analyzed *in silico* (Table 9). Moderate permeability is observed. The predicted HIA data showed that compound **3** exhibits high HIA (≥30%) whereas for compounds **1** and **2**, it is below 10%. All compounds predicted are not *P*-glycoprotein substrates or inhibitors. The data suggest that tested compounds have moderate adsorption.

**Table 9:** Prediction of ADMET Properties of Tested Compounds

	**1**	**2**	**3**
HIA	<30%	<10%	<30%
caco-2	+	+	+
CL (mL/min)	0.656	1.304	0.603
carcinogenicity (binary)	-	-	-
human intestinal absorption	+	+	+
human oral bioavailability	+	+	+
*P*-glycoprotein inhibitor	-	-	-
*P*-glycoprotein substrate	-	-	-
carcinogenicity	-	-	-
ames mutagenesis	-	-	-
blood brain barrier	-	-	-
VD (l/kg)	0.609	0.705	0.521
plasma protein binding	0.933541	0.927107573	0.983123303
CYP1A2 inhibitor	+	+	+
CYP2C19 inhibitor	+	+	+
CYP2C9 inhibitor	+	+	+
CYP2C9 substrate	-	-	-
CYP2D6 inhibitor	-	-	-
CYP2D6 substrate	-	-	-
CYP3A4 inhibitor	-	-	-
CYP3A4 substrate	-	-	-
OATP1B1 inhibitor	+	+	+
OATP1B3 inhibitor	+	+	+
OATP2B1 inhibitor	-	-	-
OCT1 inhibitor	-	-	-
OCT2 inhibitor	-	-	-
acute oral toxicity	2.539262295	2.328726769	1.877835989
mitochondrial toxicity	-	-	-
avian toxicity	-	-	-
nephrotoxicity	-	-	-
reproductive toxicity	+	+	+
respiratory toxicity	-	-	-
hepatotoxicity	+	+	+

Plasma protein binding (PPB), blood brain barrier (BBB) permeability, and volume of distribution (VD) are parameters considered to study the distribution of the tested compounds. The PPB of all compounds is about 0.9 and the BBB is negative, indicating that the distribution of these compounds is moderately distributed. The volume of distribution at steady VD values from 0.6 to 5.0 l/kg is considered moderate, and values more than 5.0 l/kg are high. The two compounds **1** and **2** are considered moderately stable compounds, while compound **3** is a highly stable compound. Regarding the total clearance (CL) value, a pharmacokinetic measurement of the plasma volume from which a substance is completely eliminated per unit of time, three measurement thresholds exist: high (>15 mL/min), average (5–15 mL/min), and low (<5 mL/min). All three compounds exhibit low clearance. Predictively, all tested compounds act as CYP1A2, CYP2C19, or CYP2C9 inhibitors. Moreover, all compounds are predicted to be OATP1B1 and OATP1B3 inhibitors but not OCT1 inhibitors. This information is valuable in understanding the potential drug–drug interactions.

These toxicity predictions suggest that tested compounds are associated with low toxicity alerts, causing low hepatotoxic and reproductive toxicity in rats.

## 4 Conclusions

This study undertakes a thorough exploration of bromophenol derivatives as potential antibacterial agents. By seamlessly integrating key aspects of chemistry, microbiology, and pharmacology, the research seeks to address significant knowledge gaps pertaining to the intricate mechanisms governing the antibacterial activity of bromophenol compounds based on their functional groups.

In conclusion, the study’s comprehensive approach, which amalgamates experimental and computational analyses, offers a solid groundwork for further research and development of bromophenol derivatives as potential antibacterial and antivirulence agents. The encouraging results against antibiotic-resistant strains, coupled with a deepened understanding of structure–activity relationships, pave the way for forthcoming efforts in drug discovery within the field of antimicrobial agents.

## Supporting Information Available

^1^H NMR (400 MHz, DMSO-*d*_6_) spectrum of 3,5-dibromo-2,6-dihydroxyacetophenone (**1**) (S1); ^1^H NMR (400 MHz, DMSO-*d*_6_) spectrum of 3-bromo-2,6-dihydroxyacetophenone (**2**) (S2); ^1^H NMR (400 MHz, DMSO-*d*_6_) spectrum of 3,5-dibromo-2,4-dihydroxyacetophenone (**3**) (S3); determination of the diameter of zone inhibition (S4); determination of the MIC value of antibiotics on PA (S5); and determination of the MIC value of antibiotics on SA (S6) (PDF)
