# Supplementary material for: Comprehensive Exploration of Bromophenol Derivatives: Promising Antibacterial Agents against SA and MRSA
Source: ACS Omega. 2024 Sep 20;9(39):40897–906. doi: 10.1021/acsomega.4c06115 (PMC11447708; doi:10.1021/acsomega.4c06115)
Supplement: Supplementary file 1 — ao4c06115_si_001.pdf [file ao4c06115_si_001.pdf]

Comprehensive Exploration of Bromophenol Derivatives: Promising Antibacterial Agents against SA and MRSA

Ta Ngoc Ly[1,*], Le My Lan[1], Ming-Yu Tsai[2], Yun-Wen Chen[3], Hsin-Yi Hung[2*].

[1]The University of Da Nang - University of Science and Technology, Danang 550000, Vietnam

[2]School of Pharmacy and Institute of Clinical Pharmacy and Pharmaceutical Sciences, College of Medicine, National Cheng Kung University, Tainan 701, Taiwan

[3]Departments of Pharmacology, College of Medicine, National Cheng Kung University, Tainan 701, Taiwan

*Corresponding author: T.N. L. email: tnly@dut.udn.vn; H-Y. H. email: z10308005@email.ncku.edu.tw

Content

S1. ^{1}H NMR (400 MHz, DMSO-*d6*) spectrum of 3,5-dibromo-2,6-dihydroxyacetophenone (**1**)

S2. ^{1}H NMR (400 MHz, DMSO-*d6*) spectrum of 3-bromo-2,6-dihydroxyacetophenone (**2**)

S3. ^{1}H NMR (400 MHz, DMSO-*d6*) spectrum of 3,5-dibromo-2,4-dihydroxyacetophenone (**3**)

S4. Determination of the diameter of zone inhibition

S5. Determine the MIC value of antibiotics on PA

S6. Determine the MIC value of antibiotics on SA

^1H NMR (400 MHz, DMSO-*d*6) spectrum of 3,5-dibromo-2,6-dihydroxyacetophenone (**1**)

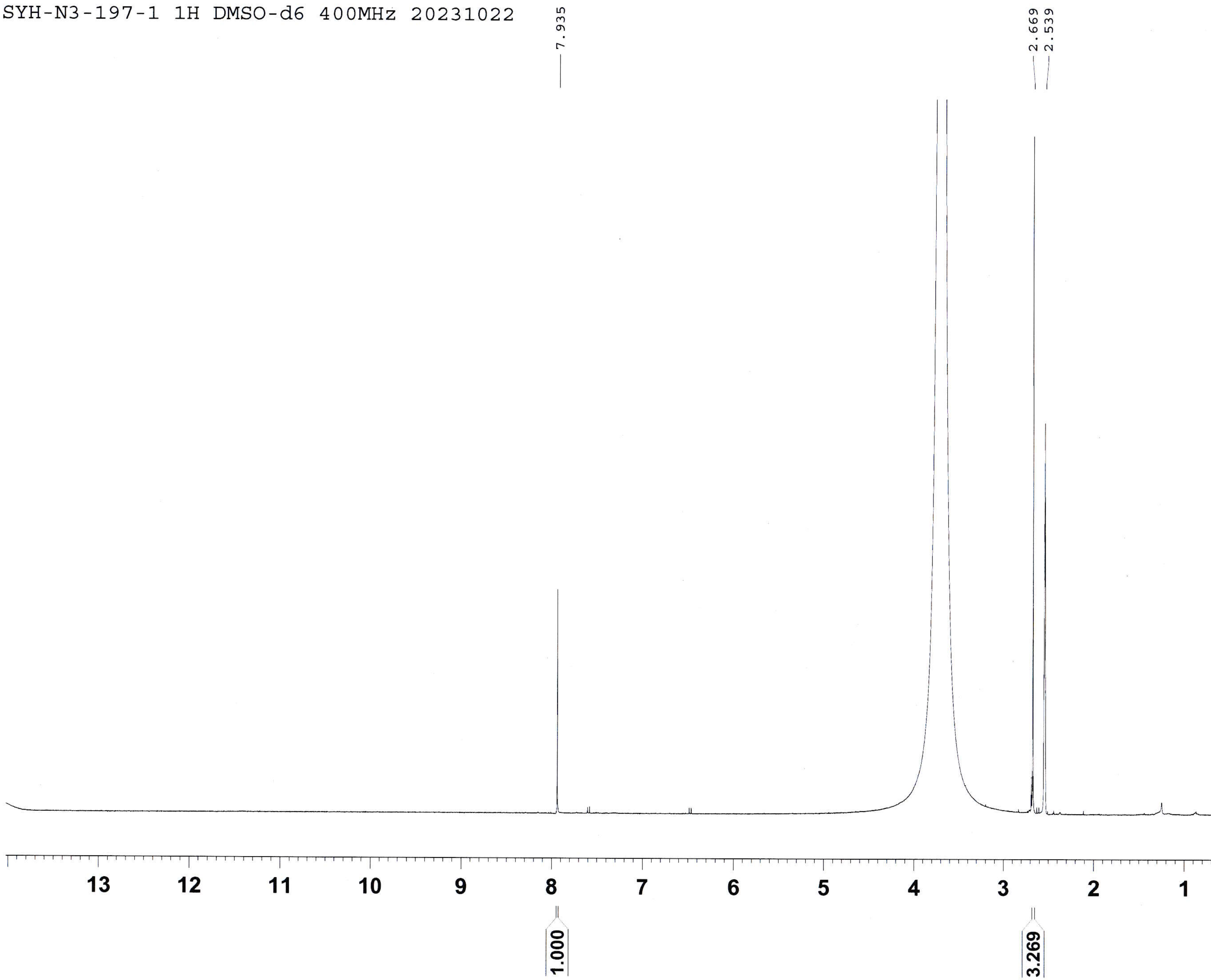

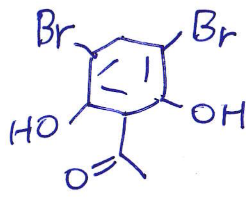

1H NMR (400 MHz, DMSO-d6) spectrum of 3-bromo-2,6-dihydroxyacetophenone (2)

SYH-N3-197-3 1H DMSO-d6 400MHz 20231022

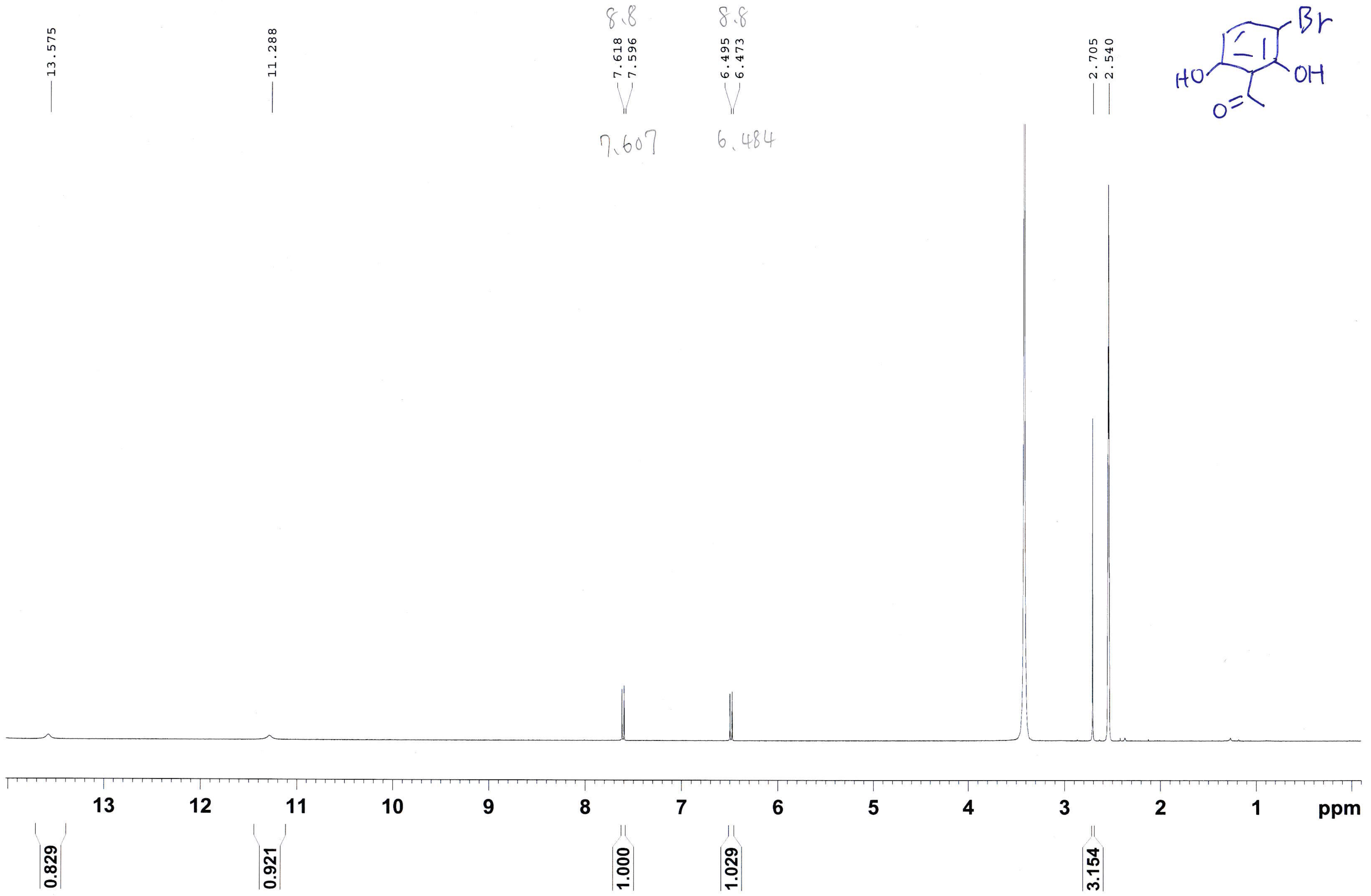

1H NMR (400 MHz, DMSO-d6) spectrum of 3,5-dibromo-2,4-dihydroxyacetophenone (3)

SYH-N3-199 1H DMSO-d6 400MHz 20231022

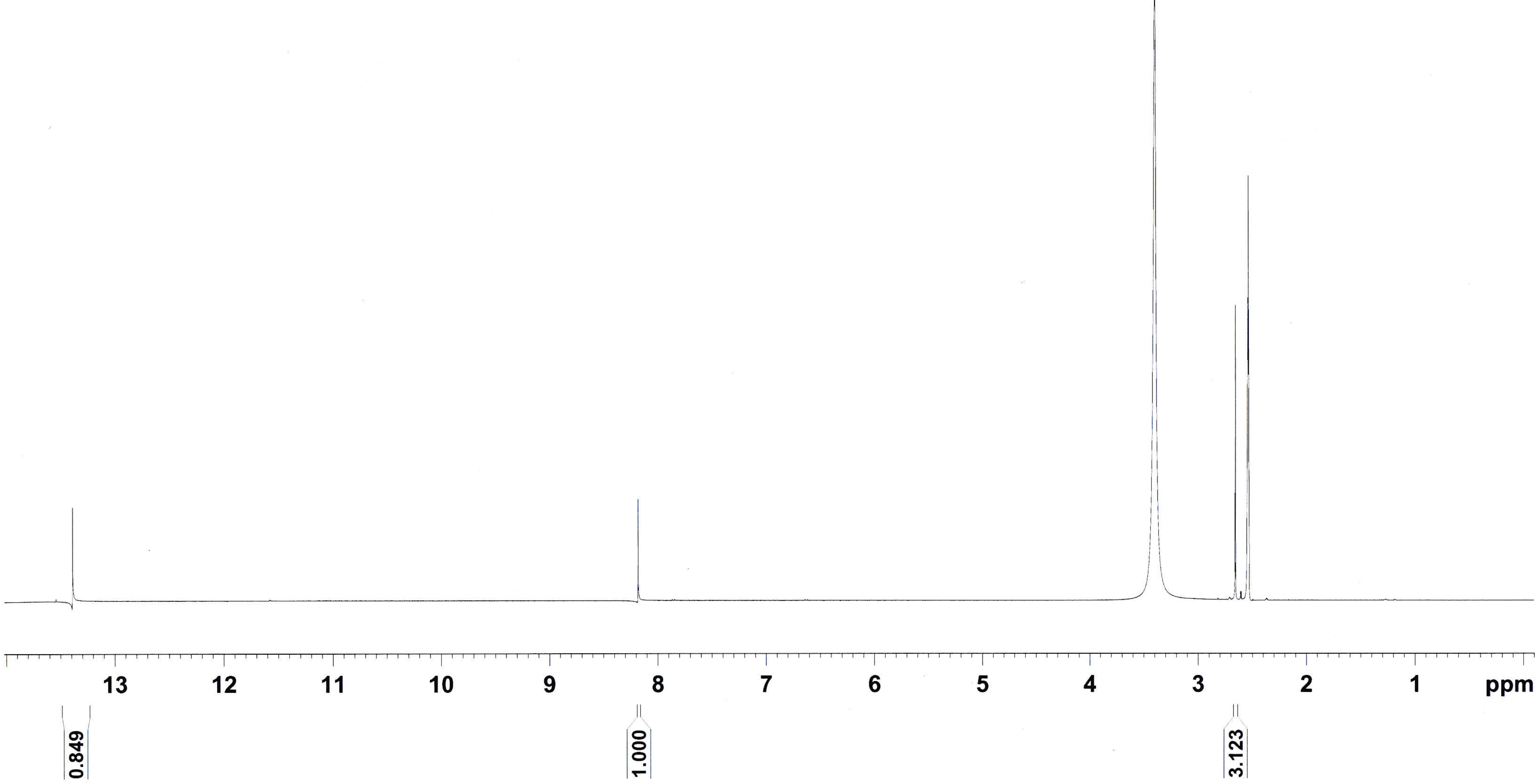

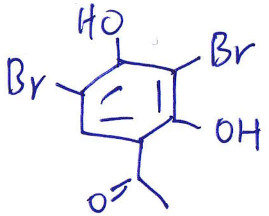

S4. Determine the diameter of zone inhhibition

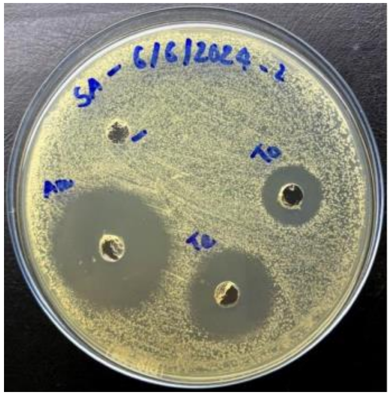

Diameter of zone inhibition on SA:
- Ampicillin: 14.5±0.5mm
- Tetracyline: 9.75 ± 0.25mm
- Tobramycin: 5.1 ± 0.1mm

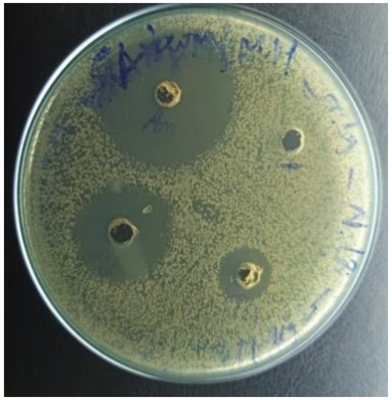

Diameter of zone inhibition on MRSA:
- Ampicillin: 15.5 ± 0.5mm
- Tetracyline: 10.25 ± 0.75mm
- Tobramycin: 5.5 ± 0.5mm

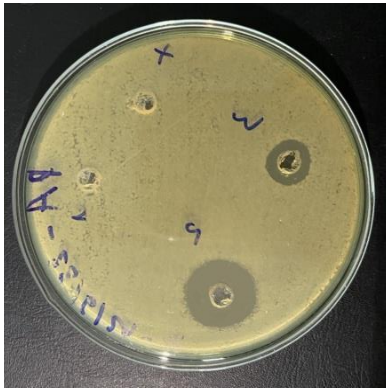

Diameter of zone inhibition on PA:
- Ampicillin:0
- Tetracyline: 10.5 ± 0.5mm
- Tobramycin: 5.5 ± 0.5mm

S5. Determine the MIC value of antibiotics on PA

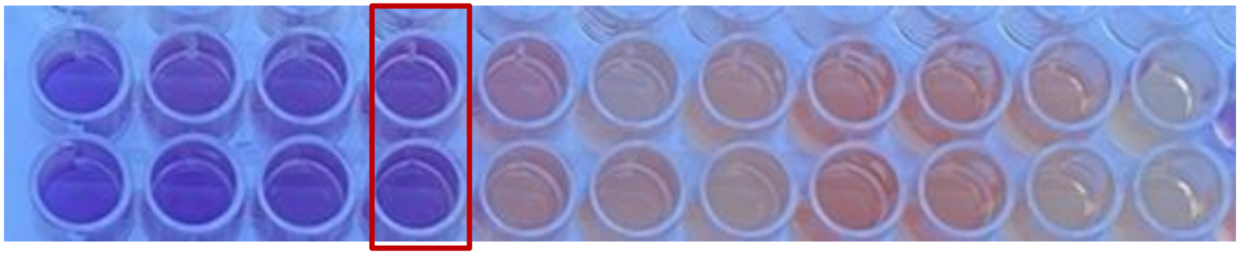

560 280 140 70 35 17.5 8.75 4.375 2.18 1.09 0.54

Tetracyline
MIC=70(μg/ml**)**

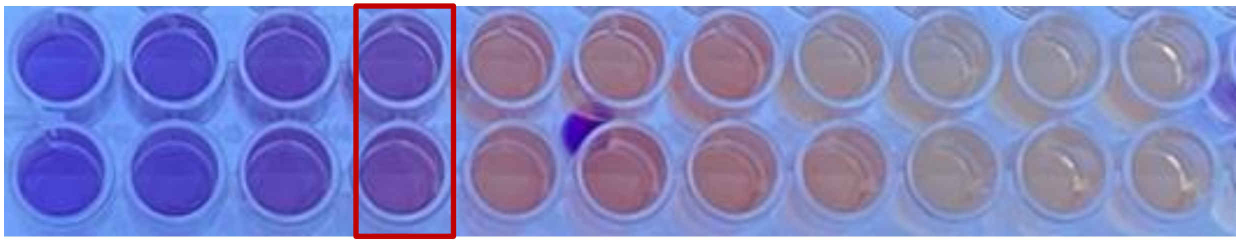

120 60 30 15 7.5 3.75 1.87 0.93 0.46 0.23 0.11

Tobramycin
MIC=15(μg/ml**)**

S6. Determine the MIC value of antibiotics on SA

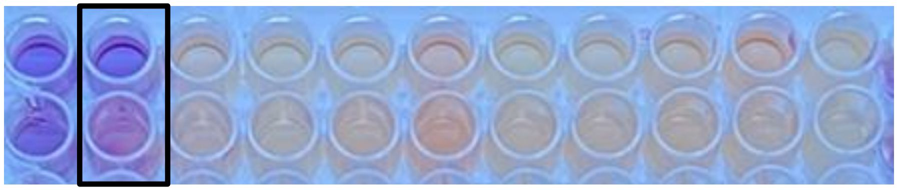

20 10 5 2.5 1.25 0.625 0.31 0.15 0.07 0.03 0.01

Ampicillin
MIC= 10(μg/ml)**)**

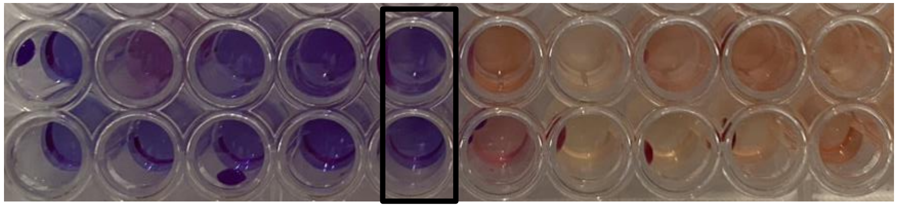

480 240 120 60 30 15 7.5 3.75 1.87 0.9

Tetracyline
MIC=30 (μg/ml)**)**

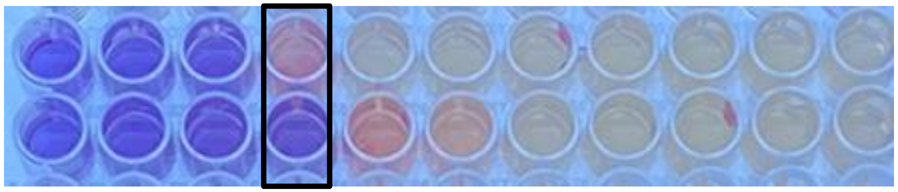

200 100 50 25 12.5 6.25 3.12 1.56 0.78 0.39 0.19

Tobramycin
MIC= 25(μg/ml)**)**
